# Managing an extension of screening intervals: Avoiding boom and bust in health care workloads

**DOI:** 10.1002/ijc.34441

**Published:** 2023-02-01

**Authors:** Francesca Pesola, Matejka Rebolj, Peter Sasieni

**Affiliations:** ^1^ Cancer Prevention Group, School of Cancer and Pharmaceutical Sciences Faculty of Life Sciences and Medicine, King's College London London UK; ^2^ Present address: Centre for Public Health and Policy, Wolfson Institute of Population Health Queen Mary University of London London UK

**Keywords:** interval extension, intervals, mass screening, workload

## Abstract

Extending screening intervals in ongoing cancer screening programmes can lead to challenging year‐on‐year variations in the number of screening tests. We explored how such variation could be diminished with a managed transition to the extended interval. We defined three extension scenarios: immediate extension for the entire target population; stepped transition by birth cohort; and gradual transition by reducing the number of available screening appointments. These were compared to a situation in which the interval remains unchanged in a demographic model covering a 15‐year period. The model was populated with observed parameters from England, a real‐world setting recommending cervical screening with 3‐year intervals at age 25‐49 and 5‐year intervals at age 50‐64. Informed by typical changes currently considered by several European programmes including the programme in England, we explored the effect on screening test numbers of an extension of the 3‐year interval to 5 years for women younger than 50. All three extension scenarios resulted in similar cumulative numbers of screening tests, which were about 30% lower compared to a situation in which the interval would remain unchanged. However, the year‐on‐year variation in the number of screening tests varied between the scenarios. This variation was around 4‐fold for the immediate scenario. In the stepped scenario, the yearly numbers could differ by around 20%, whereas in the gradual scenario they were virtually constant. A managed interval extension, transitioning different groups of the target population at different times, can substantially reduce the yearly variation in screening workload without increasing the total number of screening tests in the long term.

## Graphical abstract

What's new?

Factors such as improved testing and reductions in disease burden allow for the extension of intervals between scheduled cancer screening appointments. Unmanaged interval extension, however, can pose significant challenges for screening programs. The authors of the present study examined possible solutions for different extension scenarios, using observed parameters for cervical cancer screening in England. Analyses show that shifting subgroups of the target population to the desired extended interval at different times can help even out potentially overwhelming annual variations in screening workload. This transition can occur in the absence of increases in demand and cost over the long term.

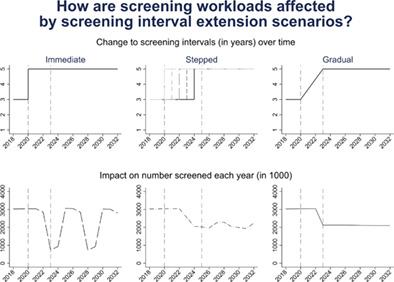

**Abbreviations**
HPV: human papillomavirus
ONS: Office for National Statistics

## 1 INTRODUCTION

Within an on‐going screening programme, improved tests, changes in service organisation, or a decreasing burden of a disease may require a longer screening interval than the interval currently in use, if cost‐effectiveness of the service is to be maintained. However, an unmanaged extension of that interval can create serious challenges due to inconsistent demands on workforce. As an example, consider a generic screening programme in which individuals are screened biennially from age 45 to 74 and the plan is to continue screening once every 4 years. Assume that there are 100 000 people in each single year of age screened each year, so that over a 2‐year cycle there are 6 million people screened. If everyone screened in 2022 and 2023 is next invited 4 years later, then in years 2024 and 2025 only the 200 000 individuals invited for the first time at ages 45 and 46, and possibly a few older people who missed an earlier screen would attend. Hence, for 2 years in each 4‐year cycle the programme would screen a couple of hundred thousand rather than 3 million people a year, creating recurrent peaks and troughs in the programme's workload.

Data from routine screening programmes confirm this. In Wales in 2013, for instance, the cervical screening interval was extended for women aged 50‐64 from 3 to 5 years as evidence showed very little benefit of more frequent screening in women regularly screened in their 30 s and 40 s.^1^ The number of screening samples from women aged 50‐64 fell by 61% from 60 678 in 2014‐2015 to 23 799 in 2017‐2018. In 2018‐2019, the numbers tested aged 50‐64 increased again to 39 154; a 65% increase compared to 2017‐2018.^2^

The challenge for any screening programme, therefore, is how to implement a new interval and redistribute the individuals examined during the old interval over the new, longer interval without causing peaks and troughs in the service's workload. In this article, we consider several solutions to this problem.

## 2 METHODS

### 2.1 General principles for screening interval extension scenarios

**FIGURE 1:**
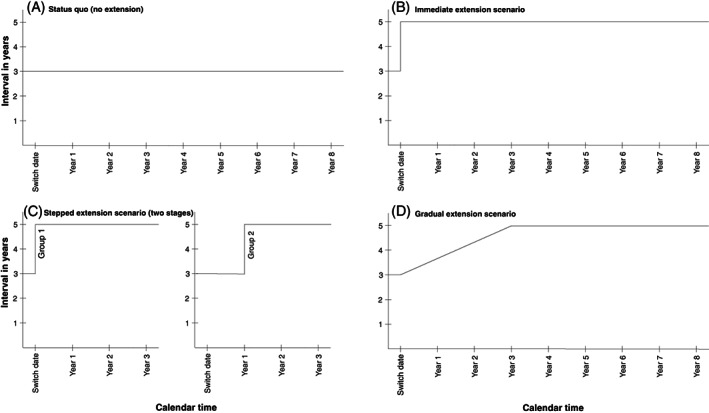
Extension of the screening interval from 3 to 5 years: comparison of the scenarios.

We explored four scenarios focusing on the interval for individuals with negative screening tests. In the first scenario, this interval remained unchanged (status quo; Figure 1A). The remaining three scenarios concerned an introduction of a longer interval (ie, immediate, stepped and gradual scenarios) and were developed as follows. We assumed that the new interval is implemented on a specific date (“switch date”) and that all primary screening after that date uses the new screening test. Women continue to be screened at 3‐year intervals after their last cytology test. In other words, their “next‐test‐due dates” are honoured until they are finally screened with HPV testing. The exact point at which the entire target population is transferred to the extended interval, however, depends on the scenario. In the immediate extension scenario, all individuals with a negative screen after the switch date are immediately assigned to a longer screening interval (Figure 1B). In the stepped extension scenario, different age groups (or birth cohorts) are moved to the extended interval at different times after the switch date (Figure 1C). Finally, in the gradual extension scenario, the number of screening appointments available each month is immediately reduced to the number that would be needed to ensure a steady state with the new interval. That change forces a gradual extension of the interval (Figure 1D).

### 2.2 Context for the analysis

To explore the effect of these interval extension scenarios on screening workload, we studied them in the context of cervical screening. In several European countries, women with negative screening cytology have been invited for screening every 3 years, although longer screening intervals have been in some cases used for women older than 50 years, for example in Denmark, Sweden, Ireland and all four UK countries. These programmes are currently being transformed by substituting cytology with testing for high‐risk human papillomavirus (HPV), which is more highly sensitive for the detection of progressive lesions and is associated with a longer lead time. Consequently, extending routine recall intervals for screen‐negative women to 5 years (with few exceptions)^3^ has been demonstrated as safe.^4^

As a simplified numerical example to illustrate the effect on screening workload, we imposed the three interval extension scenarios described above on the population of England. In England, women have been invited every 3 years at age 25‐49 and every 5 years at age 50‐64 (Supplementary Information, Figure S1A). HPV‐based primary screening was rolled out nationally in December 2019. The UK National Screening Committee recommended an extension of the screening interval for women younger than 50 from 3 to 5 years.^5^ England is a particularly suitable example for our study as the projected size of the target population (women aged 25‐64 years) is fairly stable,^6^, ^7^ which makes it easier to study extension intervals in isolation. Nevertheless, the same principles would apply in any other country introducing similar changes to its screening policy.

### 2.3 Interval extension scenarios applied to the studied context

With the invitational schedule that has been in place for cytology‐based screening in England and certain other European countries, women would be invited 12 times during their lifetime, provided they attend without a delay and have no abnormalities which would require (temporary) changes to their screening schedules. In this simplified example, a screening programme would invite nine birth cohorts younger than 50 and three birth cohorts older than 50 every year (Supplementary Information, Figure S1B).

After an implementation of the immediate interval extension scenario, the yearly number of birth cohorts invited for screening would fluctuate between 3 and 12 (Supplementary Information, Figure S1C). It may be worthwhile to note that this fluctuation would gradually diminish in the longer term, when older cohorts exit the programme and are replaced by younger cohorts screened with a 5‐year interval from their first screen onwards.

We assumed that the stepped extension scenario would be implemented in 5 years, with five blocks of birth cohorts sequentially moved to the new interval in order of seniority. Following this approach, the programme would invite between eight and 10 cohorts per year (Supplementary Information, Figure S1D). A quicker implementation would result in more variable numbers of invited birth cohorts, similar to the immediate extension scenario, whereas a slower implementation would prolong the transition period with continued screening at 3‐year intervals for some birth cohorts.

In the gradual scenario, an extension of the interval from 3 years (which implies screening of one‐third of the target population each year) to 5 years (which implies screening of one‐fifth of the population each year) would require 40% less screening capacity. The scenario would, therefore, restrict the number of available screening appointments for HPV‐negative women younger than 50 years to 60% of the numbers in the status quo scenario (this change would not affect women attending early recall; Supplementary Information, Figure S1E,F). The gradually increasing length of these intervals during the transitional phase would depend on when the next appointment slots become available and would range between 36 and 60 months. Those screened in the first month after the switch date would have a next test due date in 36.4 months. Those screened 6 months after the switch date would have an interval of 40 months until their next invitation. At 12 months the interval would be 44 months; at 24 months it would be 52 months and by 36 months it would have been extended fully to 60 months. After completion of this round, all women would be assigned next‐test‐due dates following the now standardised 5‐year interval.

### 2.4 Statistical analysis and data sources

We developed a demographic model whose structure has been described in detail elsewhere.^8^ Briefly, the model was built for the 15‐year period of 2018‐2032 and included women aged 25‐64 during that period that is, born between 1954 and 2007. We set the switch date to December 2019 when HPV testing was introduced in England (albeit without a change to the screening interval). In general, the choice of the switch date would determine the years in which any peaks and troughs would occur but should not change the relative patterns. The starting point in 2018 was chosen to allow for a comparison with the period before the switch date.

Primary outcomes of our analysis were scenario‐specific temporal trends in the yearly numbers of primary screening tests for women aged 25‐64 years. These were dependent on the numbers of women who were alive each year and at risk of developing cervical cancer, and the proportion attending screening. Each birth cohort was assigned an age‐specific screening schedule for each interval extension scenario (Supplementary Information, Figure S1A‐E). To simplify the model, we assumed that women attended in the year in which they were invited, and that their invitational schedules were not adjusted to accommodate the time needed for diagnosis and resolution of abnormalities. The resulting screening schedules were used to calculate the numbers of tests for each calendar year and birth cohort. The total numbers of screening appointments for each calendar year were then calculated by summing across all birth cohorts.

The yearly numbers of female residents aged 25‐64 in England in 2018‐2032 were retrieved from the English Office for National Statistics (ONS) population projections (Supplementary Information, Table S1).^6^, ^7^ These projections suggest a fairly stable size of the target population for cervical screening, between 14.5 and 15 million. We assumed that these women would be at risk of developing cervical cancer and invited for screening unless they had undergone a hysterectomy. The prevalence of hysterectomy in the target population was approximated by the proportion of women who had been ceased from the programme for clinical reasons (Table 1).^9^ Furthermore, we assumed that screening coverage would remain stable over time. As no changes have yet been announced, birth cohorts offered vaccination were assumed to follow the same screening schedule as older cohorts.

**TABLE 1:** Screening parameters in the model.

	Age group (years)		
Parameter	25‐29	30‐49	50‐64
Screening coverage^a^ ^,^ ^b^	70.2%	70.2%	73.6%
Target population ceased from the programme for clinical reasons^a^	0.06%	30‐34:0.15% 35‐39:0.42% 40‐44:1.14% 45‐49:3.08%	50‐54:6.19% 55‐59:9.67% 60‐64:13.88%

^a^ From the official programme statistics in England.^9^

^b^ In the last 3.5 years for women younger than 50 years, and in the last 5 years for women aged 50‐64 years (5.5‐year coverage is not routinely reported) in the (pre‐pandemic) screening year April 2019 to March 2020.

When screening intervals are extended because a more sensitive test has replaced a less sensitive one (such as when HPV testing replaces cytology), programmes may expect an increase in the number of individuals who require diagnostic evaluation. This may be more pronounced in the first (“prevalence”) round, when the new test is expected to detect not only incident lesions but also lesions that the old test could not detect.^10^, ^11^, ^12^ Additionally, in the case of cervical cancer, the proportion with abnormalities and the associated diagnostic workload is decreasing due to the highly effective HPV vaccination.^13^, ^14^ We presented the expected trends in the number of colposcopies conditional on the interval extension scenario considering both developments. We assumed that women with positive HPV tests are referred to colposcopy directly if they have abnormal cytology, and to early recall if they have negative cytology. These recommendations are shared between several cervical screening programmes internationally, although they can differ in the number of early recalls (we assumed up to two, as has been the practice across the United Kingdom). Furthermore, we used assumptions on the effect of HPV testing and vaccination on screening outcomes that were aligned with the current evidence from the literature. These accounted for the increased proportion of women with positive screening tests particularly during the first HPV‐based screening round and the likely decrease in the subsequent rounds, as well as for the differences in the effect between the bivalent (Cervarix) vs quadrivalent (Gardasil) HPV vaccination. All assumptions are explained in detail in Supplementary Information (including Table S2).

## 3 RESULTS

**FIGURE 2:**
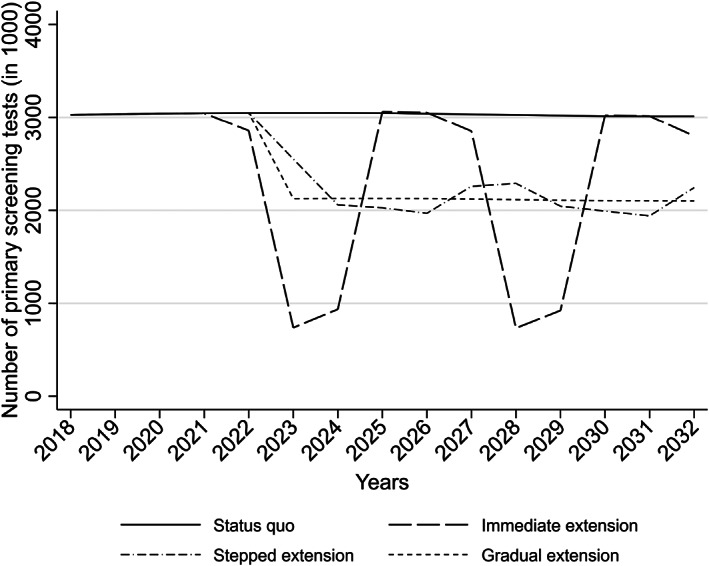
Projected yearly numbers of primary screening tests until 2032, by interval extension scenario.

Between the switch date (December 1, 2019) and the end of the analysed period (December 31, 2032), the status quo scenario would require 39.7 million primary screening tests. During the same period, each of the three interval extension scenarios would require 30.3‐30.8 million screening tests, which is 23%‐24% less than under the status quo scenario (not tabulated). These numbers included the first screening round after the switch date, during which the number of tests is expected to remain very similar across all four scenarios (Figure 2). When this round was excluded, the patterns remained similar: while the status quo scenario would require, in total, 30.3 million screening tests, the three extension scenarios would require between 21.4 and 21.6 million tests (a reduction of 29%‐30%, Table 2).

**TABLE 2:** Numbers of primary screening tests for each interval extension scenario from December 1, 2022 to December 31, 2032 that is, after the completion of the first screening round following the switch date.

Screening interval extension scenario	Minimum number in any year	Maximum number in any year	Mean number	Cumulative number	Reduction in the cumulative number
Status quo	3011	3049	3030	30 555	(Reference)
Immediate	735	3061	2119	21 369	−30%
Stepped	1939	2551	2144	21 618	−29%
Gradual	2101	2128	2123	21 409	−30%

*Note*: Absolute numbers are reported in thousands.

Despite this similarity, the yearly numbers of screening tests differed greatly between the three extension scenarios (Figure 2). The immediate extension scenario would produce recurring peaks (maximum: 3.1 million per year) and troughs (minimum: 0.7 million per year) in the numbers of screening tests, with a mean number of 2.1 million per year (Table 2). These fluctuations would diminish after 2040 and even more so after 2050, once a significant number of birth cohorts undergoing screening at the time of the switch date would have already exited the programme and new cohorts entering the screening programme will be automatically screened every 5 years (not tabulated). With the stepped extension scenario, the screening workload would fluctuate between 1.9 and 2.6 million per year, with a mean of 2.1 million. The gradual extension scenario ensured, by design, stable numbers of tests, around 2.1 million per year.

**FIGURE 3:**
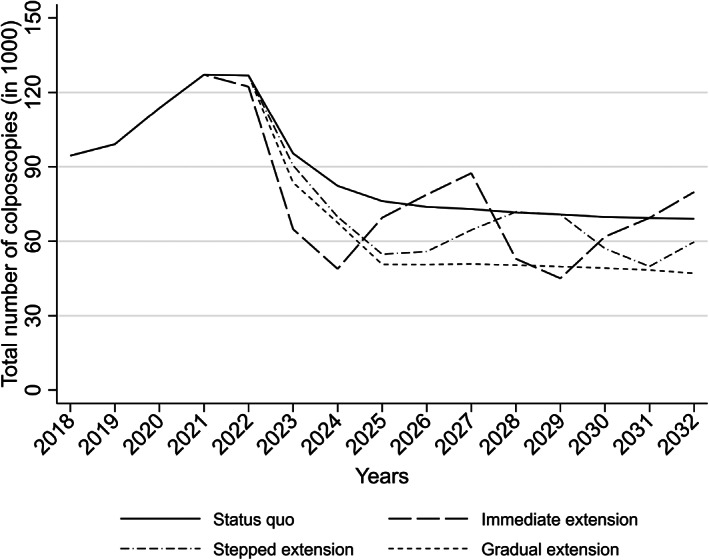
Projected yearly numbers of women referred to colposcopy until 2032, by interval extension scenario.

Figure 3 reports the expected trends in the number of women referred to colposcopy by the interval extension scenario. An initial peak is expected to be seen during the first screening round with HPV testing, particularly among women referred after early recall. As with primary tests (Figure 2), the choice of an extension scenario does not play a major role during this period. Thereafter, the patterns of peaks and troughs are expected to mirror those for primary screening tests in the sense that the stepped and gradual extension scenarios were associated with smaller year‐on‐year variations in the workload than the immediate scenario. The role of the switch from cytology to HPV testing in shaping these trends was separated from the role of HPV vaccination in Figure S2 and Table S3 (Supplementary Information). The expected patterns in the numbers of women with early recall after initially HPV‐positive, cytology‐negative screening samples were similar as for colposcopy (Supplementary Information, Figure S3; see Figure S4 for combined primary screening and early recall samples).

## 4 DISCUSSION

### 4.1 Main findings

Our analysis estimated how the volumes of health care services required to support a screening programme would vary from one year to the next following an unmanaged, immediate screening interval extension. When applying an immediate interval extension from 3 to 5 years to one part of the target population, the differences between the peak and trough workloads for the entire programme were estimated to be up to 4‐fold and to recur cyclically for several screening rounds. If, however, the same interval extension were managed by moving different subgroups to the extended interval at a different pace, the peaks and troughs could be much flatter. This could be achieved without increasing the long‐term demand for of screening tests, which is an important component of the overall cost of any screening programme.

### 4.2 Comparison with the literature and clinical significance

An implementation of a more sensitive test, which permits the extension of screening intervals, usually also changes the proportion of screened individuals who require a diagnostic evaluation (particularly so if the new test is less specific). In cervical screening, the change of the screening test has furthermore coincided with the screening of cohorts that had been offered HPV vaccination during early adolescence. Even HPV vaccination, however, appears unlikely to mitigate the cyclical changes in diagnostic workloads expected from an immediate, unmanaged, interval extension.

Cancer screening programmes typically need to provide high‐quality service with limited resources. Not having the required capacity may not only delay the scheduling of appointments and the reporting of test outcomes, but may also delay implementation of beneficial changes such as an extension of the programme across a larger target population, or an introduction of better tests.^15^, ^16^ While resource constraints are a well‐known problem, idle capacity poses its own set of organisational challenges. Temporarily diminished demand for staff, materials, or equipment due to troughs associated with, for example, an interval extension would require the kind of contracting flexibility that may not be available to many screening programmes. A possible solution to keep resources fully employed during periods with lower activity may be to reassign these resources to other tasks. During the most acute periods of the COVID‐19 pandemic, for example, many cervical screening laboratories remained in full operation by providing COVID‐19 testing.^17^ Solutions like these might not be permanent and may come at a cost not only for the delivery of the testing required for cancer screening^18^ but also in terms of a de‐skilling of the personnel and a lack of incentive to train in specialised areas.

Although both the stepped and the gradual interval extension scenarios can successfully reduce the year‐on‐year variation in the numbers of tests, there are some differences between them. The stepped scenario may allow for a slightly less sudden downscaling of the screening volumes but produces somewhat larger long‐term fluctuations than the gradual scenario, although even those were of a smaller magnitude compared to the immediate extension scenario. Potentially, another advantage of the gradual scenario over the stepped scenario is a greater degree of equality between the different groups of the target population. This is because the gradual scenario does not require a formal choice of the groups to be prioritised for an earlier switch to the longer interval, which may be important in terms of the acceptability of the change to the target population. However, it requires more capacity planning and needs to be supported by a good information system which might not be available in all screening settings.^16^

As demonstrated here, the variability in the need for screening tests after an interval extension is largely predictable. Hence, planning for these changes is essential to avoid a boom and bust of the services. HPV vaccination is already reducing the burden of cervical cancer in highly vaccinated populations,^19^, ^20^ so cost‐effective delivery of screening to vaccinated women will require a later starting age and/or a longer screening interval than for women who are unvaccinated.^21^ De‐intensification of screening of vaccinated cohorts, therefore, provides another imminent real‐life example where planning as described in our study could lessen the impact on the daily running of the service.

Furthermore, the general ideas for managed extension scenarios presented in our analysis could also be applied to a variety of situations where there is an expected shortfall of screening samples. Recently, for example, the COVID‐19 pandemic required several programmes to pause their services in the spring of 2020.^17^, ^22^ These screening providers may now expect another dip in their workloads in the spring of 2023 (if their screening interval is 3 years) or in the spring of 2025 (if their screening interval is 5 years). It is now too late to reverse this, but managed scenarios could still be put in place to tide the providers over. These scenarios could, for example, include measures such as a shorter screening interval for women due for screening in the affected years, or major public campaigns to increase screening participation.

### 4.3 Strengths and limitations

Our demographic model was informed by assumptions on the population size and its age distribution, vaccination uptake, screening behaviour and screening test characteristics observed within the same real‐life screening programme. However, it had a simplified structure. For example, it did not allow for delays in attending for screening or follow‐up procedures. In practice, slippage of (ie, heterogeneity in) actual intervals might result in a gradual flattening of the peaks and troughs over time. An Australian microsimulation model was used to investigate the impact of an interval extension from 2 to 5 years following an “immediate” extension scenario (whereas solutions to the problem of extended intervals were not investigated).^23^ Although that model was built with a much more complex structure and more detailed screening attendance assumptions, it nevertheless predicted a pattern of peaks and troughs with numbers of tests varying between 0.5 and 2 million for two decades or longer, which was consistent with our findings. Similarly, data from Wales where the screening interval was extended from 3 to 5 years for women aged 50 and over, and where attendance followed real‐world heterogeneous patterns, also indicates similar peaks and troughs.^2^ Before the COVID‐19 pandemic, furthermore, the total annual numbers of primary screening tests handled by English cervical screening laboratories were relatively stable at just under 3 million.^9^ Attendance patterns were heterogeneous, although an increasing number of women were beginning to attend at recommended intervals.^24^

## 5 CONCLUSION

Managed interval extension can avoid significant peaks and troughs in the required health care capacity without increasing the costs of a cancer screening programme. While our results were based on a model for cervical screening, it is likely that they could be generalised to screening programmes for other cancers.

## AUTHOR CONTRIBUTIONS

*Conceptualisation*: Peter Sasieni. *Literature review*: Francesca Pesola, Matejka Rebolj. *Statistical analysis*: Francesca Pesola, Peter Sasieni. *Writing (original draft)*: Francesca Pesola, Matejka Rebolj. *Writing (review and editing)*: Francesca Pesola, Matejka Rebolj, Peter Sasieni. *Decision to submit*: Francesca Pesola, Matejka Rebolj, Peter Sasieni. The work reported in the article has been performed by the authors, unless clearly specified in the text.

## FUNDING INFORMATION

Francesca Pesola was supported by Cancer Research UK (reference: C8162/A25356, awarded to Prof Peter Sasieni). Matejka Rebolj was supported by Cancer Research UK (reference: C8162/A27047, awarded to Prof Peter Sasieni). Cancer Research UK had no role in designing the study; in the collection of the data; in the writing of the manuscript; and in the decision to submit.

## CONFLICT OF INTEREST STATEMENT

Francesca Pesola reports no conflicts of interest. Matejka Rebolj reports funding from Public Health England for the epidemiological evaluation of several studies; membership in advisory groups supporting the English Cervical Screening Programme; meetings with HPV assay manufacturers; one‐time fee for a lecture from Hologic paid to the employer. Peter Sasieni received personal fees from Hologic and non‐financial support from PreventX outside the work presented in this report, and is Vice‐Director of the NIHR Policy Research Unit in Cancer Awareness, Screening and Early Diagnosis.

## Supporting information


**Data S1:** Supporting Information

## Data Availability

All data included in our study were retrieved from the published literature. Further information is available from the corresponding author upon request.
