# Supplementary material for: Managing an extension of screening intervals: Avoiding boom and bust in health care workloads
Source: Int J Cancer. 2023 Feb 1;152(10):2061–8. doi: 10.1002/ijc.34441 (PMC10952902; doi:10.1002/ijc.34441)
Supplement: Supplementary file 1 — Data S1: Supporting Information [file IJC-152-2061-s001.pdf]

# MANAGING AN EXTENSION OF SCREENING INTERVALS: THE IMPLICATIONS FOR HEALTH CARE WORKLOADS

## SUPPLEMENTARY INFORMATION

Francesca Pesola, Matejka Rebolj, Peter Sasieni

**Contents**

1. Early recall tests and colposcopies for investigation of women with positive screening tests
2. Table S1. Population projection from the Office for National Statistics, for England, females, in 2018-2032, in thousands.
3. Table S2. Colposcopy and early recall parameters in the model.
4. Table S3. Total numbers of primary screening tests, early recall tests, and women attending colposcopy (in thousands) between 1 December 2019 and 31 December 2032, by strategy of extending the screening interval for women younger than 50 years.
5. Figure S1. Birth cohorts due for screening, by calendar year and screening interval extension scenario. Drawn under the assumption that women are initially invited for screening every three years at the age of 25-49 and every five years at the age of 50-64, and that the three-year screening interval in women younger than 50 is extended to five years.
6. Figure S2. Number of women with a colposcopy depending on the primary screening test (LBC vs. HPV testing) and HPV vaccination, by calendar year and screening interval extension scenario.
7. Figure S3. Early recall tests depending on HPV vaccination, by calendar year and screening interval extension scenario.
8. Figure S4. All tests (primary and early recall combined) depending on the primary screening test (LBC vs. HPV testing) and HPV vaccination, by calendar year and screening interval extension scenario.

*Early recall tests and colposcopies for investigation of women with positive screening tests*

Follow-up of positive cervical screening tests differs by country. In England, for example, women are referred to routine recall after a negative HPV test at baseline. Those with a positive HPV test combined with borderline or more severely abnormal triage cytology are referred to colposcopy. Women with a positive HPV test combined with negative triage cytology are referred to early recall at 12 and 24 months. They are then referred to colposcopy in case of a persistently positive HPV test either following incident abnormal triage cytology at 12 months or regardless of triage cytology at 24 months. Women with a negative HPV test at early recall are referred to routine recall. Women whose primary screening test was cytology were referred to routine recall if they had negative cytology, or borderline or low-grade abnormalities combined with a negative HPV triage test. Other women were referred to colposcopy, whereas early recall was not used.

Input parameters for our demographic model were selected from the literature and describe the observations from the English HPV screening pilot (Table S2).[3-6] We assumed that HPV-positive women would attend early recall and colposcopy in the year of the referral, and that, if required, women have one colposcopy appointment. The proportions in Table S2 reflected a situation in which women younger than 50 were invited to screening every three years. For scenarios where the next invitation would be delayed to four or five years after the previous screen, we multiplied the observed proportions by 4/3 and 5/3, respectively.[7] To account for irregularly screened women, we assumed that 10% of screens in the second and later HPV rounds were from previously un- or under-screened women. These episodes were considered to lead to the same detection of abnormalities as episodes for women screened with HPV testing for the first time.

In all studied interval extension scenarios, women were assumed to have their first (prevalence-round) HPV-based screening test as soon as they become eligible for a new invitation in or after the switch date (here: December 2019), which is dependent on their birth year. For women younger than 50 who were eligible for invitations already before the switch date, this was usually three years after their last cytology-based screen. In England, however, HPV-based screening was implemented gradually through the HPV screening pilot study and the national policy to mitigate the shortage of cytoscreeners.[8,9] For these women, the first HPV test in December 2019 or later may have already been their second or third HPV screening test. Hence, we included in our model early recall tests and early recall colposcopies for 5% of the women screened with HPV testing in 2017; 15% of women screened in 2018; and 40% of the women screened between January and November 2019.

Women screened with HPV testing before the national roll-out were managed in line with the current clinical recommendations and continued to follow the usual screening intervals.

The data from the English HPV screening pilot study applied to cohorts that had not been offered HPV vaccination. For vaccinated cohorts, we estimated the detection of abnormalities leading to early recall and colposcopy referrals as described below. In England, bivalent vaccination against HPV 16/18 (Cervarix; GSK, Brentford, UK) has been offered in a routine school-based programme to girls aged 12-13 since September 2008 with an uptake of around 85%. Additionally, a catch-up campaign was offered to girls aged 14-17 between September 2008 and August 2010, with an uptake of 40-75% depending on the school cohort. In September 2012, quadrivalent vaccine (Gardasil; Merck, Kenilworth, NJ) replaced bivalent vaccine (Cervarix; GSK, Brentford, UK). In our model, we categorised birth cohorts with respect to vaccination eligibility as follows. Women born before 1 September 1990 were not offered any vaccination through a publicly organised programme and negligible numbers were vaccinated.[10,11] Bivalent catch-up vaccination was offered to cohorts born between 1 September 1990 and 31 August 1995, bivalent routine vaccination to cohorts born between 1 September 1995 and 31 August 1999, and quadrivalent routine vaccination to cohorts born on 1 September 1999 or later.

Effectiveness against abnormal screening findings achieved by the bivalent vaccine administered during the catch-up campaign was estimated from the same English HPV screening pilot study, including women attending screening for the first time at age 24-25 until the end of 2018. Here, vaccination was estimated to prevent 41% (95% CI: 36-45) of HPV infections and 49% (95% CI: 43-54) of colposcopy referrals.[6] For bivalent vaccine, Scottish screening data showed that routine vaccination was approximately 30% more effective in preventing a range of cytological and histological abnormalities than vaccination administered at an older age through the catch-up campaign.[12] Comparing the data on the effectiveness against genotype-specific persistent HPV infection (or, if not available, seroconversion) in the FUTURE I/II (Gardasil) and PATRICIA (Cervarix) randomised trials,[13-16] the effectiveness of routinely administered quadrivalent vaccination was estimated at about 76% of that which could be expected with routinely administered bivalent vaccination when applied to genotype distribution in an unvaccinated English population.[17,18]

Our demographic model followed birth cohorts which included vaccinated and unvaccinated women; hence, the observed vaccination coverage (around 56% on average during the catch-up campaign and around 85% during the routine programme)[19,20] was then used to estimate by how

much screen-detected abnormalities would decrease in each partially vaccinated birth cohort. These decreases were estimated as 28% (bivalent/catch-up), 54% (bivalent/routine) and 41% (quadrivalent/routine) for colposcopies, and 23%, 45%, and 34%, respectively, for HPV infections, either at baseline or at early recalls. Counterfactual sensitivity analyses were also conducted where the vaccination effect was set to 0, to estimate workload changes under a no vaccination scenario.

As no changes have yet been announced, birth cohorts offered vaccination were assumed to follow the same screening schedule as older cohorts.

With LBC as the primary screening test and no HPV vaccination, the projected trends for colposcopies roughly followed the projected trends for primary screening tests in all interval extension scenarios (Table S3 and Figure S2). The fluctuation continued to be most pronounced in the immediate interval extension scenario. Following the introduction of HPV testing without vaccination, the numbers of early recall tests and colposcopies were projected to increase, peaking in 2022. Thereafter, they would decrease as most women would be screened with HPV testing for the second or later time (Figures S2 and S3). Other than this prevalence peak, the patterns of peaks and troughs remained the same as expected under each scenario, with the stepped and gradual extension scenarios leading to smaller year-on-year variations than the immediate scenario (Figures S2-S4). Finally, HPV vaccination decreased the total numbers of women requiring early recall and/or colposcopy but did not further change the patterns caused by the extension of the interval.

Table S1. Population projection from the Office for National Statistics, for England, females, in 2018-2032, in thousands.[1,2]

| Age (years) | Calendar year | | | | | | | | | | | | | | | |
|---|---|---|---|---|---|---|---|---|---|---|---|---|---|---|---|---|
| | **2017** | **2018** | **2019** | **2020** | **2021** | **2022** | **2023** | **2024** | **2025** | **2026** | **2027** | **2028** | **2029** | **2030** | **2031** | **2032** |
| **25-29** | 1893 | 1880 | 1872 | 1855 | 1829 | 1806 | 1789 | 1771 | 1750 | 1725 | 1690 | 1669 | 1660 | 1667 | 1694 | 1737 |
| **30-34** | 1883 | 1898 | 1909 | 1914 | 1926 | 1932 | 1918 | 1907 | 1886 | 1858 | 1834 | 1815 | 1796 | 1776 | 1750 | 1716 |
| **35-39** | 1830 | 1872 | 1886 | 1891 | 1895 | 1902 | 1914 | 1923 | 1926 | 1937 | 1942 | 1927 | 1916 | 1895 | 1867 | 1843 |
| **40-44** | 1731 | 1704 | 1717 | 1752 | 1793 | 1840 | 1882 | 1895 | 1898 | 1902 | 1907 | 1919 | 1928 | 1931 | 1941 | 1947 |
| **45-49** | 1946 | 1920 | 1878 | 1840 | 1786 | 1738 | 1711 | 1723 | 1757 | 1797 | 1843 | 1884 | 1897 | 1900 | 1904 | 1910 |
| **50-54** | 1979 | 1984 | 1982 | 1966 | 1962 | 1946 | 1919 | 1878 | 1839 | 1785 | 1738 | 1711 | 1722 | 1757 | 1797 | 1842 |
| **55-59** | 1760 | 1810 | 1863 | 1912 | 1946 | 1964 | 1969 | 1966 | 1951 | 1946 | 1931 | 1904 | 1864 | 1826 | 1773 | 1726 |
| **60-64** | 1520 | 1551 | 1587 | 1633 | 1683 | 1735 | 1785 | 1836 | 1884 | 1917 | 1934 | 1940 | 1937 | 1922 | 1919 | 1904 |

Table S2. Colposcopy and early recall parameters in the model.

| Parameter in the model | Screening test | Age group (years) | | |
|---|---|---|---|---|
| | | 25-29 | 30-49 | 50-64 |
| **Colposcopy attended, baseline** | Cytology[a] | 8.7% | 2.7% | 1.1% |
| | HPV: First round[a] | 10.1% | 3.1% | 1.2% |
| | HPV: Second or later round[b] | 3.4% | 1.0% | 0.4% |
| **Colposcopy attended, after 12-month early recall** | HPV: First round[a] | 2.5% | 0.8% | 0.4% |
| | HPV: Second or later round[c] | 0.8% | 0.3% | 0.1% |
| **Colposcopy attended, after 24-month early recall** | HPV: First round[a] | 2.5% | 1.0% | 0.8% |
| | HPV: Second or later round[c] | 0.8% | 0.3% | 0.3% |
| **12-month early recall attended** | HPV: First round[a] | 13.8% | 6.0% | 3.6% |
| | HPV: Second or later round[d] | 6.9% | 3.0% | 1.8% |
| **24-month early recall attended** | HPV: First round[a] | 4.9% | 2.0% | 1.6% |
| | HPV: Second or later round[d] | 2.4% | 1.0% | 0.8% |

Abbreviations: HPV: human papillomavirus.

[a] Combining data on referrals to colposcopy or early recall in the first (prevalence) screening round[5] and the respective proportions of women who attended.[21]

[b] Assuming that the frequency of colposcopies is decreased by around two-thirds in the second (incidence) screening round compared with the first (prevalence) round.[3]

[c] Assuming that the ratio between baseline and early recall colposcopies is the same in the second (incidence) screening round as was observed for the first (prevalence) screening round.[3]

[d] Assuming that the frequency of women with positive HPV tests (and early recalls) is approximately halved in the second (incidence) screening round compared with the first (prevalence) screening round.[3]

Table S3. Total numbers of primary screening tests, early recall tests, and women attending colposcopy (in thousands) between 1 December 2019 and 31 December 2032, by strategy of extending the screening interval for women younger than 50 years.

| **Screening interval extension scenario** | **Primary screening tests, in thousands** | **Early recall tests, in thousands** | **Primary screening and early recall tests combined, in thousands** | **Women with colposcopy, in thousands** |
|---|---|---|---|---|
| **LBC primary screening, no HPV vaccination** | | | | |
| *Status quo* | 39,689 | 0 | 39,689 | 1307 |
| Immediate extension | 30,326 | 0 | 30,326 | 1089 |
| Stepped extension | 30,752 | 0 | 30,752 | 1093 |
| Gradual extension | 30,542 | 0 | 30,542 | 1107 |
| **HPV primary screening, no HPV vaccination** | | | | |
| *Status quo* | 39,689 | 2250 | 41,938 | 1404 |
| Immediate extension | 30,326 | 1915 | 32,241 | 1259 |
| Stepped extension | 30,752 | 2064 | 32,815 | 1270 |
| Gradual extension | 30,542 | 1908 | 32,450 | 1168 |
| **HPV primary screening, HPV vaccination** | | | | |
| *Status quo* | 39,689 | 1870 | 41,559 | 1064 |
| Immediate extension | 30,326 | 1592 | 31,918 | 963 |
| Stepped extension | 30,752 | 1721 | 32,473 | 960 |
| Gradual extension | 30,542 | 1598 | 32,141 | 880 |

Abbreviations. HPV: human papillomavirus.

Figure S1. Birth cohorts due for screening, by calendar year and screening interval extension scenario. Drawn under the assumption that women are initially invited for screening every three years at the age of 25-49 and every five years at the age of 50-64, and that the three-year screening interval in women younger than 50 is extended to five years.
Panel A. Birth cohort distribution among women targeted for screening at age 25-64, in 2018-2032.
Panel B. Screening schedule in the *status quo* scenario.
Panel C. Screening schedule in the immediate interval extension scenario.
Panel D. Screening schedule in the stepped interval extension scenario.
Panel E. Screening schedule in the gradual interval extension scenario, in yearly increments.
Panel F. Screening schedule in the gradual interval extension scenario, in monthly increments.

Figure S2. Number of women with a colposcopy depending on the primary screening test (LBC vs. HPV testing) and HPV vaccination, by calendar year and screening interval extension scenario.
Panel A. Primary screening test: LBC. HPV vaccination: No.
Panel B. Primary screening test: HPV testing. HPV vaccination: No.
Panel C. Primary screening test: HPV testing. HPV vaccination: Yes.

Figure S3. Early recall tests depending on HPV vaccination, by calendar year and screening interval extension scenario.
Panel A. Primary screening test: HPV testing. HPV vaccination: No.
Panel B. Primary screening test: HPV testing. HPV vaccination: Yes.

Figure S4. All tests (primary and early recall combined) depending on the primary screening test (LBC vs. HPV testing) and HPV vaccination, by calendar year and screening interval extension scenario.
Panel A. Primary screening test: LBC. HPV vaccination: No.
Panel B. Primary screening test: HPV testing. HPV vaccination: No.
Panel C. Primary screening test: HPV testing. HPV vaccination: Yes.

Figure S1A

| Birth cohort | 2018 | 2019 | 2020 | 2021 | 2022 | 2023 | 2024 | 2025 | 2026 | 2027 | 2028 | 2029 | 2030 | 2031 | 2032 |
|---|---|---|---|---|---|---|---|---|---|---|---|---|---|---|---|
| 2007 | | | | | | | | | | | | | | | 25 |
| 2006 | | | | | | | | | | | | | | 25 | 26 |
| 2005 | | | | | | | | | | | | | 25 | 26 | 27 |
| 2004 | | | | | | | | | | | | 25 | 26 | 27 | 28 |
| 2003 | | | | | | | | | | | 25 | 26 | 27 | 28 | 29 |
| 2002 | | | | | | | | | | 25 | 26 | 27 | 28 | 29 | 30 |
| 2001 | | | | | | | | | 25 | 26 | 27 | 28 | 29 | 30 | 31 |
| 2000 | | | | | | | | 25 | 26 | 27 | 28 | 29 | 30 | 31 | 32 |
| 1999 | | | | | | | 25 | 26 | 27 | 28 | 29 | 30 | 31 | 32 | 33 |
| 1998 | | | | | | 25 | 26 | 27 | 28 | 29 | 30 | 31 | 32 | 33 | 34 |
| 1997 | | | | | 25 | 26 | 27 | 28 | 29 | 30 | 31 | 32 | 33 | 34 | 35 |
| 1996 | | | | 25 | 26 | 27 | 28 | 29 | 30 | 31 | 32 | 33 | 34 | 35 | 36 |
| 1995 | | | 25 | 26 | 27 | 28 | 29 | 30 | 31 | 32 | 33 | 34 | 35 | 36 | 37 |
| 1994 | | 25 | 26 | 27 | 28 | 29 | 30 | 31 | 32 | 33 | 34 | 35 | 36 | 37 | 38 |
| 1993 | 25 | 26 | 27 | 28 | 29 | 30 | 31 | 32 | 33 | 34 | 35 | 36 | 37 | 38 | 39 |
| 1992 | 26 | 27 | 28 | 29 | 30 | 31 | 32 | 33 | 34 | 35 | 36 | 37 | 38 | 39 | 40 |
| 1991 | 27 | 28 | 29 | 30 | 31 | 32 | 33 | 34 | 35 | 36 | 37 | 38 | 39 | 40 | 41 |
| 1990 | 28 | 29 | 30 | 31 | 32 | 33 | 34 | 35 | 36 | 37 | 38 | 39 | 40 | 41 | 42 |
| 1989 | 29 | 30 | 31 | 32 | 33 | 34 | 35 | 36 | 37 | 38 | 39 | 40 | 41 | 42 | 43 |
| 1988 | 30 | 31 | 32 | 33 | 34 | 35 | 36 | 37 | 38 | 39 | 40 | 41 | 42 | 43 | 44 |
| 1987 | 31 | 32 | 33 | 34 | 35 | 36 | 37 | 38 | 39 | 40 | 41 | 42 | 43 | 44 | 45 |
| 1986 | 32 | 33 | 34 | 35 | 36 | 37 | 38 | 39 | 40 | 41 | 42 | 43 | 44 | 45 | 46 |
| 1985 | 33 | 34 | 35 | 36 | 37 | 38 | 39 | 40 | 41 | 42 | 43 | 44 | 45 | 46 | 47 |
| 1984 | 34 | 35 | 36 | 37 | 38 | 39 | 40 | 41 | 42 | 43 | 44 | 45 | 46 | 47 | 48 |
| 1983 | 35 | 36 | 37 | 38 | 39 | 40 | 41 | 42 | 43 | 44 | 45 | 46 | 47 | 48 | 49 |
| 1982 | 36 | 37 | 38 | 39 | 40 | 41 | 42 | 43 | 44 | 45 | 46 | 47 | 48 | 49 | 50 |
| 1981 | 37 | 38 | 39 | 40 | 41 | 42 | 43 | 44 | 45 | 46 | 47 | 48 | 49 | 50 | 51 |
| 1980 | 38 | 39 | 40 | 41 | 42 | 43 | 44 | 45 | 46 | 47 | 48 | 49 | 50 | 51 | 52 |
| 1979 | 39 | 40 | 41 | 42 | 43 | 44 | 45 | 46 | 47 | 48 | 49 | 50 | 51 | 52 | 53 |
| 1978 | 40 | 41 | 42 | 43 | 44 | 45 | 46 | 47 | 48 | 49 | 50 | 51 | 52 | 53 | 54 |
| 1977 | 41 | 42 | 43 | 44 | 45 | 46 | 47 | 48 | 49 | 50 | 51 | 52 | 53 | 54 | 55 |
| 1976 | 42 | 43 | 44 | 45 | 46 | 47 | 48 | 49 | 50 | 51 | 52 | 53 | 54 | 55 | 56 |
| 1975 | 43 | 44 | 45 | 46 | 47 | 48 | 49 | 50 | 51 | 52 | 53 | 54 | 55 | 56 | 57 |
| 1974 | 44 | 45 | 46 | 47 | 48 | 49 | 50 | 51 | 52 | 53 | 54 | 55 | 56 | 57 | 58 |
| 1973 | 45 | 46 | 47 | 48 | 49 | 50 | 51 | 52 | 53 | 54 | 55 | 56 | 57 | 58 | 59 |
| 1972 | 46 | 47 | 48 | 49 | 50 | 51 | 52 | 53 | 54 | 55 | 56 | 57 | 58 | 59 | 60 |
| 1971 | 47 | 48 | 49 | 50 | 51 | 52 | 53 | 54 | 55 | 56 | 57 | 58 | 59 | 60 | 61 |
| 1970 | 48 | 49 | 50 | 51 | 52 | 53 | 54 | 55 | 56 | 57 | 58 | 59 | 60 | 61 | 62 |
| 1969 | 49 | 50 | 51 | 52 | 53 | 54 | 55 | 56 | 57 | 58 | 59 | 60 | 61 | 62 | 63 |
| 1968 | 50 | 51 | 52 | 53 | 54 | 55 | 56 | 57 | 58 | 59 | 60 | 61 | 62 | 63 | 64 |
| 1967 | 51 | 52 | 53 | 54 | 55 | 56 | 57 | 58 | 59 | 60 | 61 | 62 | 63 | 64 | |
| 1966 | 52 | 53 | 54 | 55 | 56 | 57 | 58 | 59 | 60 | 61 | 62 | 63 | 64 | | |
| 1965 | 53 | 54 | 55 | 56 | 57 | 58 | 59 | 60 | 61 | 62 | 63 | 64 | | | |
| 1964 | 54 | 55 | 56 | 57 | 58 | 59 | 60 | 61 | 62 | 63 | 64 | | | | |
| 1963 | 55 | 56 | 57 | 58 | 59 | 60 | 61 | 62 | 63 | 64 | | | | | |
| 1962 | 56 | 57 | 58 | 59 | 60 | 61 | 62 | 63 | 64 | | | | | | |
| 1961 | 57 | 58 | 59 | 60 | 61 | 62 | 63 | 64 | | | | | | | |
| 1960 | 58 | 59 | 60 | 61 | 62 | 63 | 64 | | | | | | | | |
| 1959 | 59 | 60 | 61 | 62 | 63 | 64 | | | | | | | | | |
| 1958 | 60 | 61 | 62 | 63 | 64 | | | | | | | | | | |
| 1957 | 61 | 62 | 63 | 64 | | | | | | | | | | | |
| 1956 | 62 | 63 | 64 | | | | | | | | | | | | |
| 1955 | 63 | 64 | | | | | | | | | | | | | |
| 1954 | 64 | | | | | | | | | | | | | | |

Calendar year

Figure S1B

| Birth cohort | 2018 | 2019 | 2020 | 2021 | 2022 | 2023 | 2024 | 2025 | 2026 | 2027 | 2028 | 2029 | 2030 | 2031 | 2032 |
|---|---|---|---|---|---|---|---|---|---|---|---|---|---|---|---|
| 2007 | | | | | | | | | | | | | | | 3 |
| 2006 | | | | | | | | | | | | | | 3 | |
| 2005 | | | | | | | | | | | | | 3 | | |
| 2004 | | | | | | | | | | | | 3 | | | 3 |
| 2003 | | | | | | | | | | | 3 | | | 3 | |
| 2002 | | | | | | | | | | 3 | | | 3 | | |
| 2001 | | | | | | | | | 3 | | | 3 | | | 3 |
| 2000 | | | | | | | | 3 | | | 3 | | | 3 | |
| 1999 | | | | | | | 3 | | | 3 | | | 3 | | |
| 1998 | | | | | | 3 | | | 3 | | | 3 | | | 3 |
| 1997 | | | | | 3 | | | 3 | | | 3 | | | 3 | |
| 1996 | | | | 3 | | | 3 | | | 3 | | | 3 | | |
| 1995 | | | 3 | | | 3 | | | 3 | | | 3 | | | 3 |
| 1994 | | 3 | | | 3 | | | 3 | | | 3 | | | 3 | |
| 1993 | 3 | | | 3 | | | 3 | | | 3 | | | 3 | | |
| 1992 | | | 3 | | | 3 | | | 3 | | | 3 | | | 3 |
| 1991 | | 3 | | | 3 | | | 3 | | | 3 | | | 3 | |
| 1990 | 3 | | | 3 | | | 3 | | | 3 | | | 3 | | |
| 1989 | | | 3 | | | 3 | | | 3 | | | 3 | | | 3 |
| 1988 | | 3 | | | 3 | | | 3 | | | 3 | | | 3 | |
| 1987 | 3 | | | 3 | | | 3 | | | 3 | | | 3 | | |
| 1986 | | | 3 | | | 3 | | | 3 | | | 3 | | | 3 |
| 1985 | | 3 | | | 3 | | | 3 | | | 3 | | | 3 | |
| 1984 | 3 | | | 3 | | | 3 | | | 3 | | | 3 | | |
| 1983 | | | 3 | | | 3 | | | 3 | | | 3 | | | 3 |
| 1982 | | 3 | | | 3 | | | 3 | | | 3 | | | 3 | |
| 1981 | 3 | | | 3 | | | 3 | | | 3 | | | 3 | | |
| 1980 | | | 3 | | | 3 | | | 3 | | | 3 | | | 5 |
| 1979 | | 3 | | | 3 | | | 3 | | | 3 | | | 5 | |
| 1978 | 3 | | | 3 | | | 3 | | | 3 | | | 5 | | |
| 1977 | | | 3 | | | 3 | | | 3 | | | 5 | | | |
| 1976 | | 3 | | | 3 | | | 3 | | | 5 | | | | |
| 1975 | 3 | | | 3 | | | 3 | | | 5 | | | | | 5 |
| 1974 | | | 3 | | | 3 | | | 5 | | | | | 5 | |
| 1973 | | 3 | | | 3 | | | 5 | | | | | 5 | | |
| 1972 | 3 | | | 3 | | | 5 | | | | | 5 | | | |
| 1971 | | | 3 | | | 5 | | | | | 5 | | | | |
| 1970 | | 3 | | | 5 | | | | | 5 | | | | | L |
| 1969 | 3 | | | 5 | | | | | 5 | | | | | L | |
| 1968 | | | 5 | | | | | 5 | | | | | L | | |
| 1967 | | 5 | | | | | 5 | | | | | L | | | |
| 1966 | 5 | | | | | 5 | | | | | L | | | | |
| 1965 | | | | | 5 | | | | | L | | | | | |
| 1964 | | | | 5 | | | | | L | | | | | | |
| 1963 | | | 5 | | | | | L | | | | | | | |
| 1962 | | 5 | | | | | L | | | | | | | | |
| 1961 | 5 | | | | | L | | | | | | | | | |
| 1960 | | | | | L | | | | | | | | | | |
| 1959 | | | | L | | | | | | | | | | | |
| 1958 | | | L | | | | | | | | | | | | |
| 1957 | | L | | | | | | | | | | | | | |
| 1956 | L | | | | | | | | | | | | | | |
| 1955 | | | | | | | | | | | | | | | |
| 1954 | | | | | | | | | | | | | | | |

Calendar year

| | 2018 | 2019 | 2020 | 2021 | 2022 | 2023 | 2024 | 2025 | 2026 | 2027 | 2028 | 2029 | 2030 | 2031 | 2032 |
|---|---|---|---|---|---|---|---|---|---|---|---|---|---|---|---|
| Cohorts 25-49 | 9 | 9 | 9 | 9 | 9 | 9 | 9 | 9 | 9 | 9 | 9 | 9 | 9 | 9 | 9 |
| Cohorts 50-64 | 3 | 3 | 3 | 3 | 3 | 3 | 3 | 3 | 3 | 3 | 3 | 3 | 3 | 3 | 3 |
| All cohorts | 12 | 12 | 12 | 12 | 12 | 12 | 12 | 12 | 12 | 12 | 12 | 12 | 12 | 12 | 12 |

Legend.
Numbers: next test due date advice given at screening, in years.
"L": last screening test for the birth cohort.

Figure S1C

| Birth cohort | 2018 | 2019 | 2020 | 2021 | 2022 | 2023 | 2024 | 2025 | 2026 | 2027 | 2028 | 2029 | 2030 | 2031 | 2032 |
|---|---|---|---|---|---|---|---|---|---|---|---|---|---|---|---|
| 2007 | | | | | | | | | | | | | | | 5 |
| 2006 | | | | | | | | | | | | | | 5 | |
| 2005 | | | | | | | | | | | | | 5 | | |
| 2004 | | | | | | | | | | | | 5 | | | |
| 2003 | | | | | | | | | | | 5 | | | | |
| 2002 | | | | | | | | | | 5 | | | | | 5 |
| 2001 | | | | | | | | | 5 | | | | | 5 | |
| 2000 | | | | | | | | 5 | | | | | 5 | | |
| 1999 | | | | | | | 5 | | | | | 5 | | | |
| 1998 | | | | | | 5 | | | | | 5 | | | | |
| 1997 | | | | | 5 | | | | | 5 | | | | | 5 |
| 1996 | | | | 5 | | | | | 5 | | | | | 5 | |
| 1995 | | | 5 | | | | | 5 | | | | | 5 | | |
| 1994 | | 3 | | | 5 | | | | | 5 | | | | | 5 |
| 1993 | 3 | | | 5 | | | | | 5 | | | | | 5 | |
| 1992 | | | 5 | | | | | 5 | | | | | 5 | | |
| 1991 | | 3 | | | 5 | | | | | 5 | | | | | 5 |
| 1990 | 3 | | | 5 | | | | | 5 | | | | | 5 | |
| 1989 | | | 5 | | | | | 5 | | | | | 5 | | |
| 1988 | | 3 | | | 5 | | | | | 5 | | | | | 5 |
| 1987 | 3 | | | 5 | | | | | 5 | | | | | 5 | |
| 1986 | | | 5 | | | | | 5 | | | | | 5 | | |
| 1985 | | 3 | | | 5 | | | | | 5 | | | | | 5 |
| 1984 | 3 | | | 5 | | | | | 5 | | | | | 5 | |
| 1983 | | | 5 | | | | | 5 | | | | | 5 | | |
| 1982 | | 3 | | | 5 | | | | | 5 | | | | | 5 |
| 1981 | 3 | | | 5 | | | | | 5 | | | | | 5 | |
| 1980 | | | 5 | | | | | 5 | | | | | 5 | | |
| 1979 | | 3 | | | 5 | | | | | 5 | | | | | 5 |
| 1978 | 3 | | | 5 | | | | | 5 | | | | | 5 | |
| 1977 | | | 5 | | | | | 5 | | | | | 5 | | |
| 1976 | | 3 | | | 5 | | | | | 5 | | | | | 5 |
| 1975 | 3 | | | 5 | | | | | 5 | | | | | 5 | |
| 1974 | | | 5 | | | | | 5 | | | | | 5 | | |
| 1973 | | 3 | | | 5 | | | | | 5 | | | | | 5 |
| 1972 | 3 | | | 5 | | | | | 5 | | | | | 5 | |
| 1971 | | | 5 | | | | | 5 | | | | | 5 | | |
| 1970 | | 3 | | | 5 | | | | | 5 | | | | | L |
| 1969 | 3 | | | 5 | | | | | 5 | | | | | L | |
| 1968 | | | 5 | | | | | 5 | | | | | L | | |
| 1967 | | 5 | | | | | 5 | | | | | L | | | |
| 1966 | 5 | | | | | 5 | | | | | L | | | | |
| 1965 | | | | | 5 | | | | | L | | | | | |
| 1964 | | | | 5 | | | | | L | | | | | | |
| 1963 | | | 5 | | | | | L | | | | | | | |
| 1962 | | 5 | | | | | L | | | | | | | | |
| 1961 | 5 | | | | | L | | | | | | | | | |
| 1960 | | | | | L | | | | | | | | | | |
| 1959 | | | | L | | | | | | | | | | | |
| 1958 | | | L | | | | | | | | | | | | |
| 1957 | | L | | | | | | | | | | | | | |
| 1956 | L | | | | | | | | | | | | | | |
| 1955 | | | | | | | | | | | | | | | |
| 1954 | | | | | | | | | | | | | | | |

Calendar year

| | 2018 | 2019 | 2020 | 2021 | 2022 | 2023 | 2024 | 2025 | 2026 | 2027 | 2028 | 2029 | 2030 | 2031 | 2032 |
|---|---|---|---|---|---|---|---|---|---|---|---|---|---|---|---|
| Cohorts 25-49 | 9 | 9 | 9 | 9 | 9 | 1 | 1 | 8 | 8 | 8 | 2 | 2 | 7 | 7 | 7 |
| Cohorts 50-64 | 3 | 3 | 3 | 3 | 3 | 2 | 2 | 4 | 4 | 4 | 1 | 1 | 5 | 5 | 5 |
| All cohorts | 12 | 12 | 12 | 12 | 12 | 3 | 3 | 12 | 12 | 12 | 3 | 3 | 12 | 12 | 12 |

Legend.
Numbers: next test due date advice given at screening, in years.
"L": last screening test for the birth cohort.

Figure S1D

| Birth cohort | 2018 | 2019 | 2020 | 2021 | 2022 | 2023 | 2024 | 2025 | 2026 | 2027 | 2028 | 2029 | 2030 | 2031 | 2032 |
|---|---|---|---|---|---|---|---|---|---|---|---|---|---|---|---|
| 2007 | | | | | | | | | | | | | | | 5 |
| 2006 | | | | | | | | | | | | | | 5 | |
| 2005 | | | | | | | | | | | | | 5 | | |
| 2004 | | | | | | | | | | | | 5 | | | |
| 2003 | | | | | | | | | | | 5 | | | | |
| 2002 | | | | | | | | | | 5 | | | | | 5 |
| 2001 | | | | | | | | | 5 | | | | | 5 | |
| 2000 | | | | | | | | 5 | | | | | 5 | | |
| 1999 | | | | | | | 5 | | | | | 5 | | | |
| 1998 | | | | | | 3 | | | 5 | | | | | 5 | |
| 1997 | | | | | 3 | | | 5 | | | | | 5 | | |
| 1996 | | | | 3 | | | 5 | | | | | 5 | | | |
| 1995 | | | 3 | | | 5 | | | | | 5 | | | | |
| 1994 | | 3 | | | 3 | | | 5 | | | | | 5 | | |
| 1993 | 3 | | | 3 | | | 5 | | | | | 5 | | | |
| 1992 | | | 3 | | | 5 | | | | | 5 | | | | |
| 1991 | | 3 | | | 3 | | | 5 | | | | | 5 | | |
| 1990 | 3 | | | 3 | | | 5 | | | | | 5 | | | |
| 1989 | | | 3 | | | 5 | | | | | 5 | | | | |
| 1988 | | 3 | | | 5 | | | | | 5 | | | | | 5 |
| 1987 | 3 | | | 3 | | | 5 | | | | | 5 | | | |
| 1986 | | | 3 | | | 5 | | | | | 5 | | | | |
| 1985 | | 3 | | | 5 | | | | | 5 | | | | | |
| 1984 | 3 | | | 3 | | | 5 | | | | | 5 | | | |
| 1983 | | | 3 | | | 5 | | | | | 5 | | | | |
| 1982 | | 3 | | | 5 | | | | | 5 | | | | | 5 |
| 1981 | 3 | | | 5 | | | | | 5 | | | | | 5 | |
| 1980 | | | 3 | | | 5 | | | | | 5 | | | | |
| 1979 | | 3 | | | 5 | | | | | 5 | | | | | 5 |
| 1978 | 3 | | | 5 | | | | | 5 | | | | | 5 | |
| 1977 | | | 3 | | | 5 | | | | | 5 | | | | |
| 1976 | | 3 | | | 5 | | | | | 5 | | | | | 5 |
| 1975 | 3 | | | 5 | | | | | 5 | | | | | 5 | |
| 1974 | | | 5 | | | | | 5 | | | | | 5 | | |
| 1973 | | 3 | | | 5 | | | | | 5 | | | | | 5 |
| 1972 | 3 | | | 5 | | | | | 5 | | | | | 5 | |
| 1971 | | | 5 | | | | | 5 | | | | | 5 | | |
| 1970 | | 3 | | | 5 | | | | | 5 | | | | | L |
| 1969 | 3 | | | 5 | | | | | 5 | | | | | L | |
| 1968 | | | 5 | | | | | 5 | | | | | L | | |
| 1967 | | 5 | | | | | 5 | | | | | L | | | |
| 1966 | 5 | | | | | 5 | | | | | L | | | | |
| 1965 | | | | | 5 | | | | | L | | | | | |
| 1964 | | | | 5 | | | | | L | | | | | | |
| 1963 | | | 5 | | | | | L | | | | | | | |
| 1962 | | 5 | | | | | L | | | | | | | | |
| 1961 | 5 | | | | | L | | | | | | | | | |
| 1960 | | | | | L | | | | | | | | | | |
| 1959 | | | | L | | | | | | | | | | | |
| 1958 | | | L | | | | | | | | | | | | |
| 1957 | | L | | | | | | | | | | | | | |
| 1956 | L | | | | | | | | | | | | | | |
| 1955 | | | | | | | | | | | | | | | |
| 1954 | | | | | | | | | | | | | | | |

Calendar year

| | 2018 | 2019 | 2020 | 2021 | 2022 | 2023 | 2024 | 2025 | 2026 | 2027 | 2028 | 2029 | 2030 | 2031 | 2032 |
|---|---|---|---|---|---|---|---|---|---|---|---|---|---|---|---|
| Cohorts 25-49 | 9 | 9 | 9 | 9 | 9 | 8 | 6 | 4 | 4 | 5 | 7 | 7 | 5 | 3 | 4 |
| Cohorts 50-64 | 3 | 3 | 3 | 3 | 3 | 2 | 2 | 4 | 4 | 4 | 2 | 1 | 3 | 5 | 5 |
| All cohorts | 12 | 12 | 12 | 12 | 12 | 10 | 8 | 8 | 8 | 9 | 9 | 8 | 8 | 8 | 9 |

Legend.
Numbers: next test due date advice given at screening, in years.
"L": last screening test for the birth cohort.
Cells marked gray: calendar year in which an extended screening interval begins to be implemented for a given birth cohort.

Figure S1E

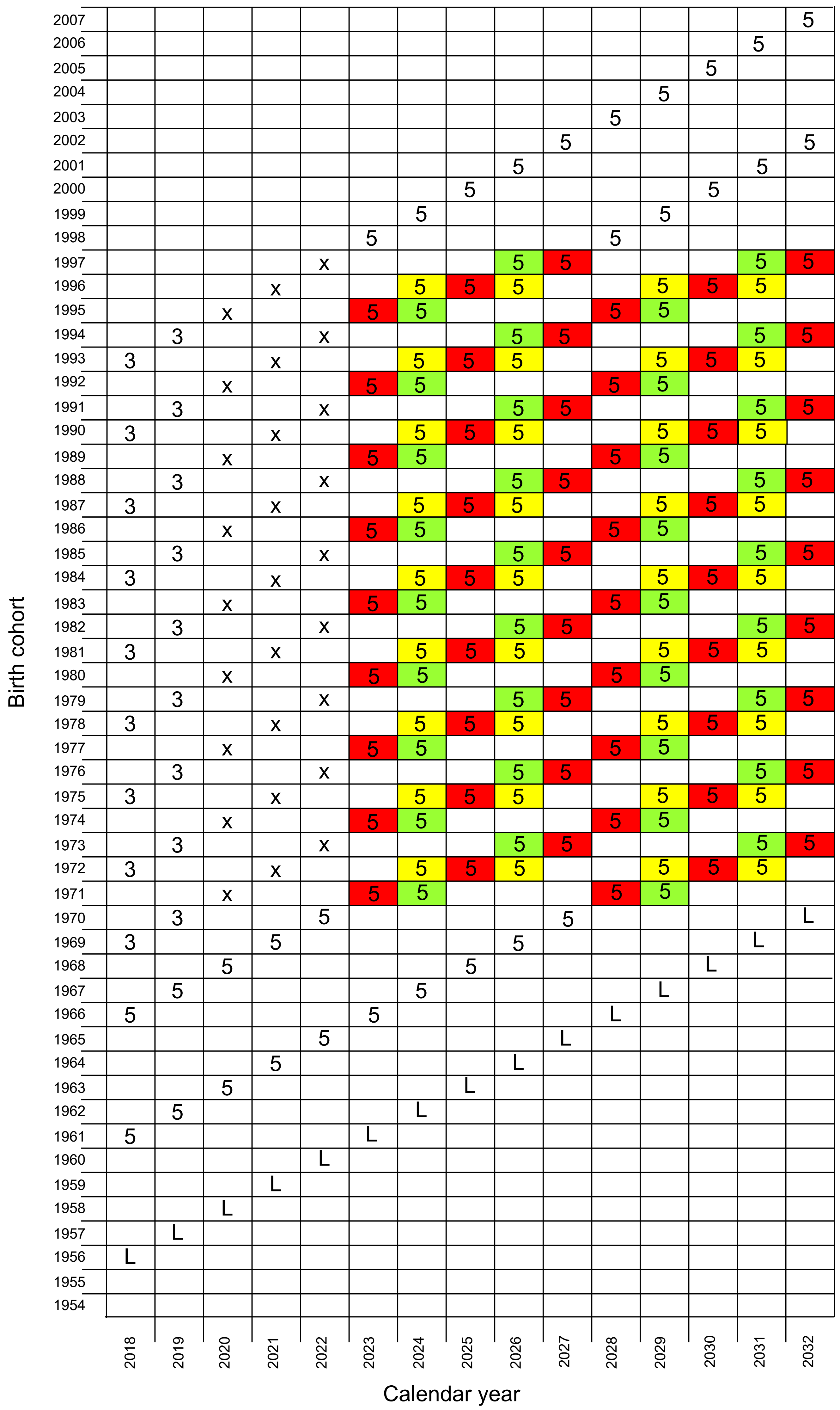


| | 2018 | 2019 | 2020 | 2021 | 2022 | 2023 | 2024 | 2025 | 2026 | 2027 | 2028 | 2029 | 2030 | 2031 | 2032 |
|---|---|---|---|---|---|---|---|---|---|---|---|---|---|---|---|
| Cohorts 25-49 | 9 | 9 | 9 | 9 | 9 | 5.8 | 5.4 | 5.2 | 5.2 | 5.2 | 5.6 | 5.6 | 5.6 | 5.4 | 5.0 |
| Cohorts 50-64 | 3 | 3 | 3 | 3 | 3 | 2.6 | 3.0 | 3.2 | 3.2 | 3.2 | 2.8 | 2.8 | 2.8 | 3.0 | 3.4 |
| All cohorts | 12 | 12 | 12 | 12 | 12 | 8.4 | 8.4 | 8.4 | 8.4 | 8.4 | 8.4 | 8.4 | 8.4 | 8.4 | 8.4 |

Legend.
Numbers: next test due date advice given at screening, in years.
"x": women are given a variable next test due date advice, ranging from 3 to 5 years.
"L": last screening test for the birth cohort.
Cells marked red: 60% of the birth cohort.
Cells marked green: 40% of the birth cohort.
Cells marked yellow: 20% of the birth cohort.

Figure S1F

**Screening interval following the implementation round (round 2)**

**Interval extension implementation round (round 1)**

| | | Year 4 | | | | | | | | | | | | Year 5 | | | | | | | | | | | | Year 6 | | | | | | | | | | | | Year 7 | | | | | | | | | | | | Year 8 | | | | | | | | | | | | |
|---|---|---|---|---|---|---|---|---|---|---|---|---|---|---|---|---|---|---|---|---|---|---|---|---|---|---|---|---|---|---|---|---|---|---|---|---|---|---|---|---|---|---|---|---|---|---|---|---|---|---|---|---|---|---|---|---|---|---|---|---|---|---|
| | | 37 | 38 | 39 | 40 | 41 | 42 | 43 | 44 | 45 | 46 | 47 | 48 | 49 | 50 | 51 | 52 | 53 | 54 | 55 | 56 | 57 | 58 | 59 | 60 | 61 | 62 | 63 | 64 | 65 | 66 | 67 | 68 | 69 | 70 | 71 | 72 | 73 | 74 | 75 | 76 | 77 | 78 | 79 | 80 | 81 | 82 | 83 | 84 | 85 | 86 | 87 | 88 | 89 | 90 | 91 | 92 | 93 | 94 | 95 | 96 | Average interval in months |
| Year 1 | 1 | 60 | 40 | | | | | | | | | | | | | | | | | | | | | | | | | | | | | | | | | | | | | | | | | | | | | | | | | | | | | | | | | | | 36.4 |
| | 2 | | 20 | 60 | 20 | | | | | | | | | | | | | | | | | | | | | | | | | | | | | | | | | | | | | | | | | | | | | | | | | | | | | | | | | 37.0 |
| | 3 | | | | 40 | 60 | | | | | | | | | | | | | | | | | | | | | | | | | | | | | | | | | | | | | | | | | | | | | | | | | | | | | | | | 37.6 |
| | 4 | | | | | | 60 | 40 | | | | | | | | | | | | | | | | | | | | | | | | | | | | | | | | | | | | | | | | | | | | | | | | | | | | | | 38.4 |
| | 5 | | | | | | | 20 | 60 | 20 | | | | | | | | | | | | | | | | | | | | | | | | | | | | | | | | | | | | | | | | | | | | | | | | | | | | 39.0 |
| | 6 | | | | | | | | | 40 | 60 | | | | | | | | | | | | | | | | | | | | | | | | | | | | | | | | | | | | | | | | | | | | | | | | | | | 39.6 |
| | 7 | | | | | | | | | | | 60 | 40 | | | | | | | | | | | | | | | | | | | | | | | | | | | | | | | | | | | | | | | | | | | | | | | | | 40.4 |
| | 8 | | | | | | | | | | | | 20 | 60 | 20 | | | | | | | | | | | | | | | | | | | | | | | | | | | | | | | | | | | | | | | | | | | | | | | 41.0 |
| | 9 | | | | | | | | | | | | | | 40 | 60 | | | | | | | | | | | | | | | | | | | | | | | | | | | | | | | | | | | | | | | | | | | | | | 41.6 |
| | 10 | | | | | | | | | | | | | | | | 60 | 40 | | | | | | | | | | | | | | | | | | | | | | | | | | | | | | | | | | | | | | | | | | | | 42.4 |
| | 11 | | | | | | | | | | | | | | | | | 20 | 60 | 20 | | | | | | | | | | | | | | | | | | | | | | | | | | | | | | | | | | | | | | | | | | 43.0 |
| | 12 | | | | | | | | | | | | | | | | | | | 40 | 60 | | | | | | | | | | | | | | | | | | | | | | | | | | | | | | | | | | | | | | | | | 43.6 |
| Year 2 | 13 | | | | | | | | | | | | | | | | | | | | | 60 | 40 | | | | | | | | | | | | | | | | | | | | | | | | | | | | | | | | | | | | | | | 44.4 |
| | 14 | | | | | | | | | | | | | | | | | | | | | | 20 | 60 | 20 | | | | | | | | | | | | | | | | | | | | | | | | | | | | | | | | | | | | | 45.0 |
| | 15 | | | | | | | | | | | | | | | | | | | | | | | | 40 | 60 | | | | | | | | | | | | | | | | | | | | | | | | | | | | | | | | | | | | 45.6 |
| | 16 | | | | | | | | | | | | | | | | | | | | | | | | | | 60 | 40 | | | | | | | | | | | | | | | | | | | | | | | | | | | | | | | | | | 46.4 |
| | 17 | | | | | | | | | | | | | | | | | | | | | | | | | | | 20 | 60 | 20 | | | | | | | | | | | | | | | | | | | | | | | | | | | | | | | | 47.0 |
| | 18 | | | | | | | | | | | | | | | | | | | | | | | | | | | | | 40 | 60 | | | | | | | | | | | | | | | | | | | | | | | | | | | | | | | 47.6 |
| | 19 | | | | | | | | | | | | | | | | | | | | | | | | | | | | | | | 60 | 40 | | | | | | | | | | | | | | | | | | | | | | | | | | | | | 48.4 |
| | 20 | | | | | | | | | | | | | | | | | | | | | | | | | | | | | | | | 20 | 60 | 20 | | | | | | | | | | | | | | | | | | | | | | | | | | | 49.0 |
| | 21 | | | | | | | | | | | | | | | | | | | | | | | | | | | | | | | | | | 40 | 60 | | | | | | | | | | | | | | | | | | | | | | | | | | 49.6 |
| | 22 | | | | | | | | | | | | | | | | | | | | | | | | | | | | | | | | | | | | 60 | 40 | | | | | | | | | | | | | | | | | | | | | | | | | 50.4 |
| | 23 | | | | | | | | | | | | | | | | | | | | | | | | | | | | | | | | | | | | | 20 | 60 | 20 | | | | | | | | | | | | | | | | | | | | | | | 51.0 |
| | 24 | | | | | | | | | | | | | | | | | | | | | | | | | | | | | | | | | | | | | | | 40 | 60 | | | | | | | | | | | | | | | | | | | | | | | 51.6 |
| Year 3 | 25 | | | | | | | | | | | | | | | | | | | | | | | | | | | | | | | | | | | | | | | | | 60 | 40 | | | | | | | | | | | | | | | | | | | | | 52.4 |
| | 26 | | | | | | | | | | | | | | | | | | | | | | | | | | | | | | | | | | | | | | | | | | 20 | 60 | 20 | | | | | | | | | | | | | | | | | | | 53.0 |
| | 27 | | | | | | | | | | | | | | | | | | | | | | | | | | | | | | | | | | | | | | | | | | | | 40 | 60 | | | | | | | | | | | | | | | | | | | 53.6 |
| | 28 | | | | | | | | | | | | | | | | | | | | | | | | | | | | | | | | | | | | | | | | | | | | | | 60 | 40 | | | | | | | | | | | | | | | | | 54.4 |
| | 29 | | | | | | | | | | | | | | | | | | | | | | | | | | | | | | | | | | | | | | | | | | | | | | | 20 | 60 | 20 | | | | | | | | | | | | | | | 55.0 |
| | 30 | | | | | | | | | | | | | | | | | | | | | | | | | | | | | | | | | | | | | | | | | | | | | | | | | 40 | 60 | | | | | | | | | | | | | | | 55.6 |
| | 31 | | | | | | | | | | | | | | | | | | | | | | | | | | | | | | | | | | | | | | | | | | | | | | | | | | | 60 | 40 | | | | | | | | | | | | | 56.4 |
| | 32 | | | | | | | | | | | | | | | | | | | | | | | | | | | | | | | | | | | | | | | | | | | | | | | | | | | | 20 | 60 | 20 | | | | | | | | | | | 57.0 |
| | 33 | | | | | | | | | | | | | | | | | | | | | | | | | | | | | | | | | | | | | | | | | | | | | | | | | | | | | | 40 | 60 | | | | | | | | | | | 57.6 |
| | 34 | | | | | | | | | | | | | | | | | | | | | | | | | | | | | | | | | | | | | | | | | | | | | | | | | | | | | | | | 60 | 40 | | | | | | | | | 58.4 |
| | 35 | | | | | | | | | | | | | | | | | | | | | | | | | | | | | | | | | | | | | | | | | | | | | | | | | | | | | | | | | 20 | 60 | 20 | | | | | | | | 59.0 |
| | 36 | | | | | | | | | | | | | | | | | | | | | | | | | | | | | | | | | | | | | | | | | | | | | | | | | | | | | | | | | | | 40 | 60 | | | | | | | 59.6 |

Legend.
Women are assigned the next test due date in months 1-36 (when they are screened in the interval extension implementation round, "round 1") for the earliest point when there are available screening capacities in the following round ("round 2"). The capacities in round 2 are capped at 60% of the volume that was available in round 1 and the previous rounds. After round 1, the next test due date increases from 3 to 5 years in monthly intervals.
Red numbers: percent of the cohort screened in a particular month in round 1 that is referred to routine recall in round 2 with the given next test due date (screening interval).

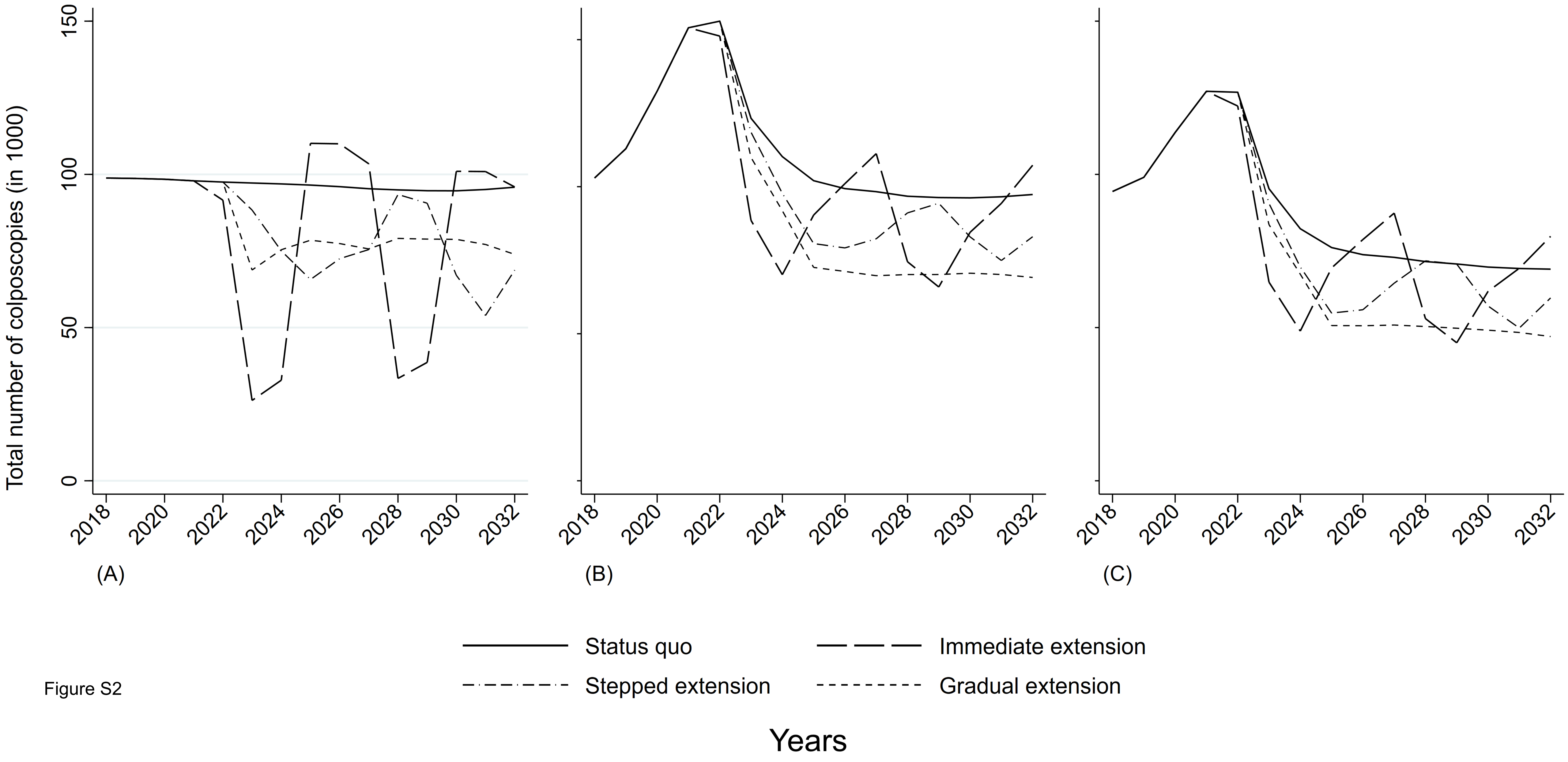
Total number of colposcopies (in 1000)
150
100
50
0
2018
2020
2022
2024
2026
2028
2030
2032
(A)
(B)
(C)
Status quo
Immediate extension
Stepped extension
Gradual extension
Years

Figure S2

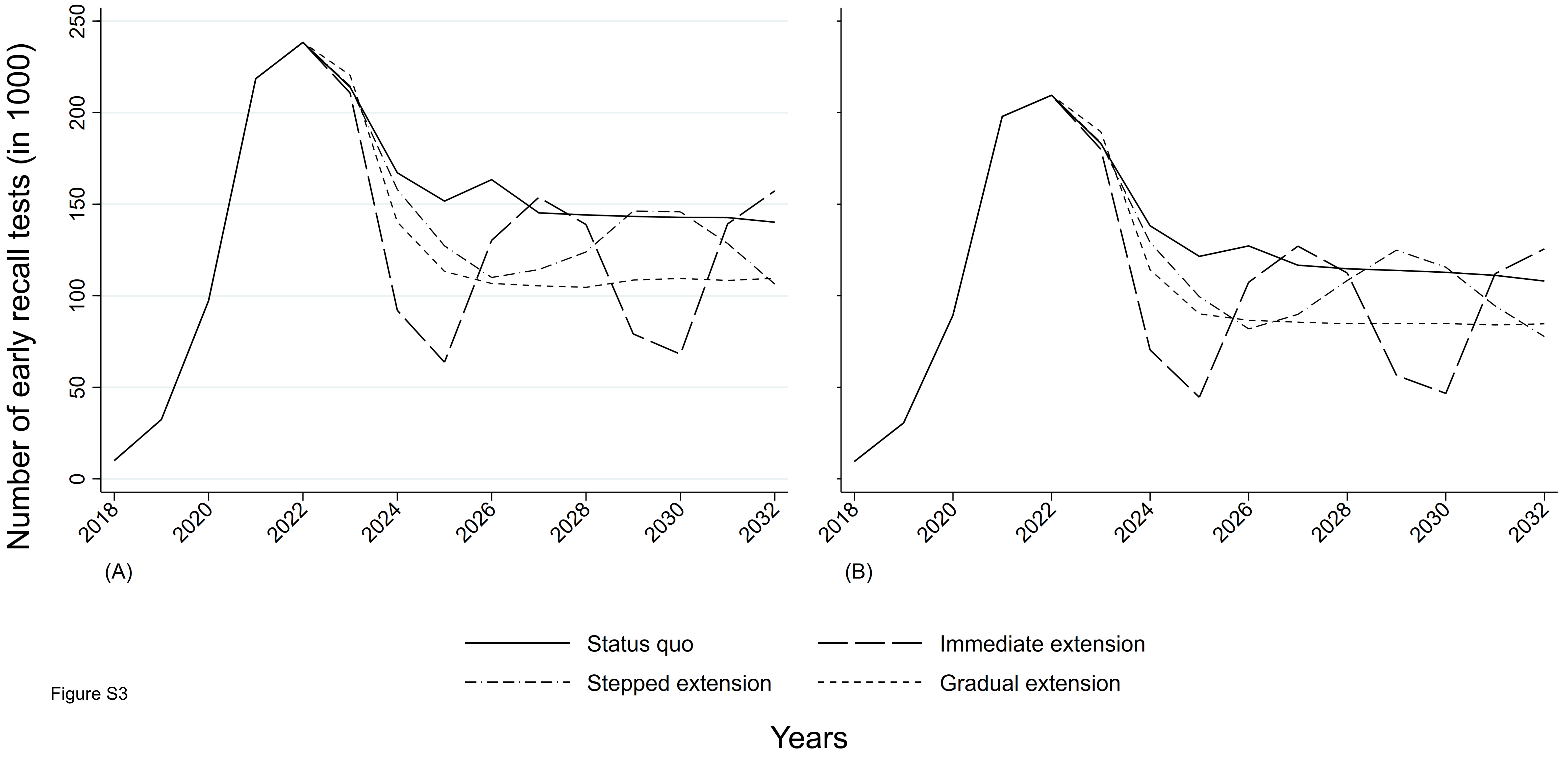

Number of early recall tests (in 1000)
0
50
100
150
200
250
2018
2020
2022
2024
2026
2028
2030
2032
(A)
(B)
Status quo
Immediate extension
Stepped extension
Gradual extension
Years


Figure S3

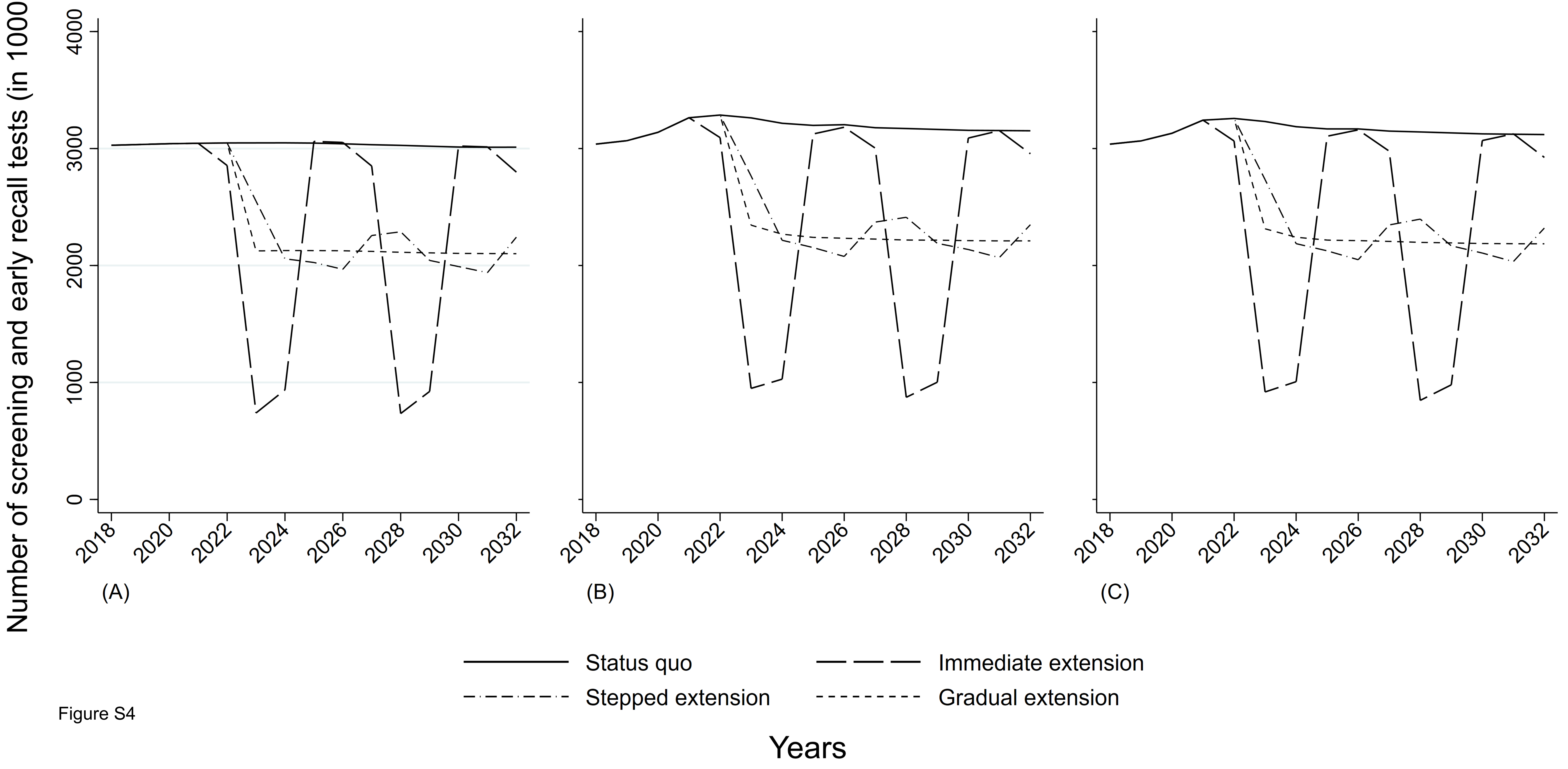

Number of screening and early recall tests (in 1000)
0
1000
2000
3000
4000
2018
2020
2022
2024
2026
2028
2030
2032
(A)
(B)
(C)
Status quo
Immediate extension
Stepped extension
Gradual extension
Years


Figure S4

## REFERENCES

1. Office for National Statistics. Estimates of the population for the UK, England and Wales, Scotland and Northern Ireland. URL: https://www.ons.gov.uk/peoplepopulationandcommunity/populationandmigration/populationestimates/datasets/populationestimatesforukenglandandwalesscotlandandnorthernireland. Last accessed: 29 September 2022.

2. Office for National Statistics. National population projections table of contents. URL: https://www.ons.gov.uk/peoplepopulationandcommunity/populationandmigration/populationprojections/datasets/2014basednationalpopulationprojectionstableofcontents. Last accessed: 22 September 2022.

3. Rebolj M, Rimmer J, Denton K, Tidy J, Mathews C, Ellis K, Smith J, Evans C, Giles T, Frew V, Tyler X, Sargent A, Parker J, Holbrook M, Hunt K, Tidbury P, Levine T, Smith D, Patnick J, Stubbs R, Moss S, Kitchener H. Primary cervical screening with high risk human papillomavirus testing: observational study. BMJ 2019; 364: l240

4. Green LI, Mathews CS, Waller J, Kitchener H, Rebolj M. Attendance at early recall and colposcopy in routine cervical screening with human papillomavirus testing. Int J Cancer 2021; 148: 1850-1857

5. Rebolj M, Mathews CS, Pesola F, Cuschieri K, Denton K, Kitchener H. Age-specific outcomes from the first round of HPV screening in unvaccinated women: Observational study from the English cervical screening pilot. BJOG 2022; 129: 1278-1288

6. Rebolj M, Pesola F, Mathews C, Mesher D, Soldan K, Kitchener H. The impact of catch-up bivalent human papillomavirus vaccination on cervical screening outcomes: an observational study from the English HPV primary screening pilot. Br J Cancer 2022; 127: 278-287

7. Ronco G, van Ballegooijen M, Becker N, Chil A, Fender M, Giubilato P, Kurtinaitis J, Lancucki L, Lynge E, Morais A, O'Reilly M, Sparen P, Suteu O, Rebolj M, Veerus P, Primic Zakelj M, Anttila A. Process performance of cervical screening programmes in Europe. Eur J Cancer 2009; 45: 2659-2670

8. Rimmer J. New guidance to help cervical screening providers reduce cytology backlogs. URL: https://phescreening.blog.gov.uk/2017/12/18/new-guidance-to-help-cervical-screening-providers-reduce-cytology-backlogs/. Last accessed: 28 September 2022.

9. Richards M, Report of the independent review of adult screening programmes in England (Publication reference 01089). URL: https://www.england.nhs.uk/wp-content/uploads/2019/02/report-of-the-independent-review-of-adult-screening-programme-in-england.pdf. Last accessed: 28 September 2022.

10. Mesher D, Panwar K, Thomas SL, Beddows S, Soldan K. Continuing reductions in HPV 16/18 in a population with high coverage of bivalent HPV vaccination in England: an ongoing cross-sectional study. BMJ Open 2016; 6: e009915

11. Cameron RL, Kavanagh K, Pan J, Love J, Cuschieri K, Robertson C, Ahmed S, Palmer T, Pollock KG. Human Papillomavirus Prevalence and Herd Immunity after Introduction of Vaccination Program, Scotland, 2009-2013. Emerg Infect Dis 2016; 22: 56-64

12. Palmer T, Wallace L, Pollock KG, Cuschieri K, Robertson C, Kavanagh K, Cruickshank M. Prevalence of cervical disease at age 20 after immunisation with bivalent HPV vaccine at age 12-13 in Scotland: retrospective population study. BMJ 2019; 365: l1161

13. Wheeler CM, Castellsague X, Garland SM, Szarewski A, Paavonen J, Naud P, Salmeron J, Chow SN, Apter D, Kitchener H, Teixeira JC, Skinner SR, Jaisamrarn U, Limson G, Romanowski B, Aoki FY, Schwarz TF, Poppe WA, Bosch FX, Harper DM, Huh W, Hardt K, Zahaf T, Descamps D, Struyf F, Dubin G, Lehtinen M. Cross-protective efficacy of HPV-16/18 AS04-adjuvanted vaccine against cervical infection and precancer caused by non-vaccine oncogenic HPV types: 4-year end-of-study analysis of the randomised, double-blind PATRICIA trial. Lancet Oncology 2012; 13: 100-110

14. Lehtinen M, Paavonen J, Wheeler CM, Jaisamrarn U, Garland SM, Castellsague X, Skinner SR, Apter D, Naud P, Salmeron J, Chow SN, Kitchener H, Teixeira JC, Hedrick J, Limson G, Szarewski A, Romanowski B, Aoki FY, Schwarz TF, Poppe WA, De Carvalho NS, Germar MJ, Peters K, Mindel A, De Sutter P, Bosch FX, David MP, Descamps D, Struyf F, Dubin G. Overall efficacy of HPV-16/18 AS04-adjuvanted vaccine against grade 3 or greater cervical intraepithelial neoplasia: 4-year end-of-study analysis of the randomised, double-blind PATRICIA trial. Lancet Oncology 2012; 13: 89-99

15. Brown DR, Kjaer SK, Sigurdsson K, Iversen OE, Hernandez-Avila M, Wheeler CM, Perez G, Koutsky LA, Tay EH, Garcia P, Ault KA, Garland SM, Leodolter S, Olsson SE, Tang GW, Ferris DG, Paavonen J, Steben M, Bosch FX, Dillner J, Joura EA, Kurman RJ, Majewski S, Muñoz N, Myers ER, Villa LL, Taddeo FJ, Roberts C, Tadesse A, Bryan J, Lupinacci LC, Giacoletti KE, Sings HL, James M, Hesley TM, Barr E. The impact of quadrivalent human papillomavirus (HPV; types 6, 11, 16, and 18) L1 virus-like particle vaccine on infection and disease due to oncogenic nonvaccine HPV types in generally HPV-naive women aged 16-26 years. J Infect Dis 2009; 199: 926-935

16. Joura EA, Kjaer SK, Wheeler CM, Sigurdsson K, Iversen OE, Hernandez-Avila M, Perez G, Brown DR, Koutsky LA, Tay EH, Garcia P, Ault KA, Garland SM, Leodolter S, Olsson SE, Tang GW, Ferris DG, Paavonen J, Lehtinen M, Steben M, Bosch X, Dillner J, Kurman RJ, Majewski S, Muñoz N, Myers ER, Villa LL, Taddeo FJ, Roberts C, Tadesse A, Bryan J, Lupinacci LC, Giacoletti KE, Lu S, Vuocolo S, Hesley TM, Haupt RM, Barr E. HPV antibody levels and clinical efficacy following administration of a prophylactic quadrivalent HPV vaccine. Vaccine 2008; 26: 6844-6851

17. Sargent A, Bailey A, Almonte M, Turner A, Thomson C, Peto J, Desai M, Mather J, Moss S, Roberts C, Kitchener HC. Prevalence of type-specific HPV infection by age and grade of cervical cytology: data from the ARTISTIC trial. Br J Cancer 2008; 98: 1704-1709

18. Kitchener HC, Almonte M, Gilham C, Dowie R, Stoykova B, Sargent A, Roberts C, Desai M, Peto J. ARTISTIC: a randomised trial of human papillomavirus (HPV) testing in primary cervical screening. Health TechnolAssess 2009; 13: 1-150

19. Public Health England, Human Papillomavirus (HPV) Vaccine Coverage in England, 2008/09 to 2013/14. A review of the full six years of the three-dose schedule. URL: https://assets.publishing.service.gov.uk/government/uploads/system/uploads/attachment_data/file/774074/HPV_Vaccine_Coverage_in_England_200809_to_201314.pdf. Last accessed: 28 September 2022.

20. Public Health England. Human papillomavirus (HPV) vaccination coverage in adolescent females and males in England: academic year 2019 to 2020. Health Protection Report Volume 14 Number 19. URL: https://www.gov.uk/government/publications/hpv-vaccination-coverage-in-adolescent-females-and-males-in-england-2019-to-2020. Last accessed: 28 September 2022.

21. Green LI, Mathews CS, Waller J, Kitchener H, Rebolj M. Attendance at early recall and colposcopy in routine cervical screening with human papillomavirus testing. Int J Cancer 2021; 148: 1850-1857
